# Supplementary material for: Genetic targeting of myelinated primary afferent neurons using a new NefhCreERT2 knock-in mouse
Source: Sci Rep. 2025 Mar 29;15:10890. doi: 10.1038/s41598-025-95874-2 (PMC11954935; doi:10.1038/s41598-025-95874-2)
Supplement: Supplementary file 1 — Supplementary Material 1. [file 41598_2025_95874_MOESM1_ESM.pdf]

Supplementary information

Genetic targeting of myelinated primary afferent neurons using a new $Nefh^{CreERT2}$ **knock-in mouse**

John CY Chen[1], Lech Kaczmarczyk[1], Felipe Meira de Faria[2], Marcin Szczot[2], Walker S Jackson[1], Max Larsson[1,*]

[1]Department of Biomedical and Clinical Sciences, Division of Cell- and Neurobiology, Linköping University, S-581 85 Linköping, Sweden

[2]Department of Biomedical and Clinical Sciences, Center for Social and Affective Neuroscience, Linköping University, S-581 85 Linköping, Sweden

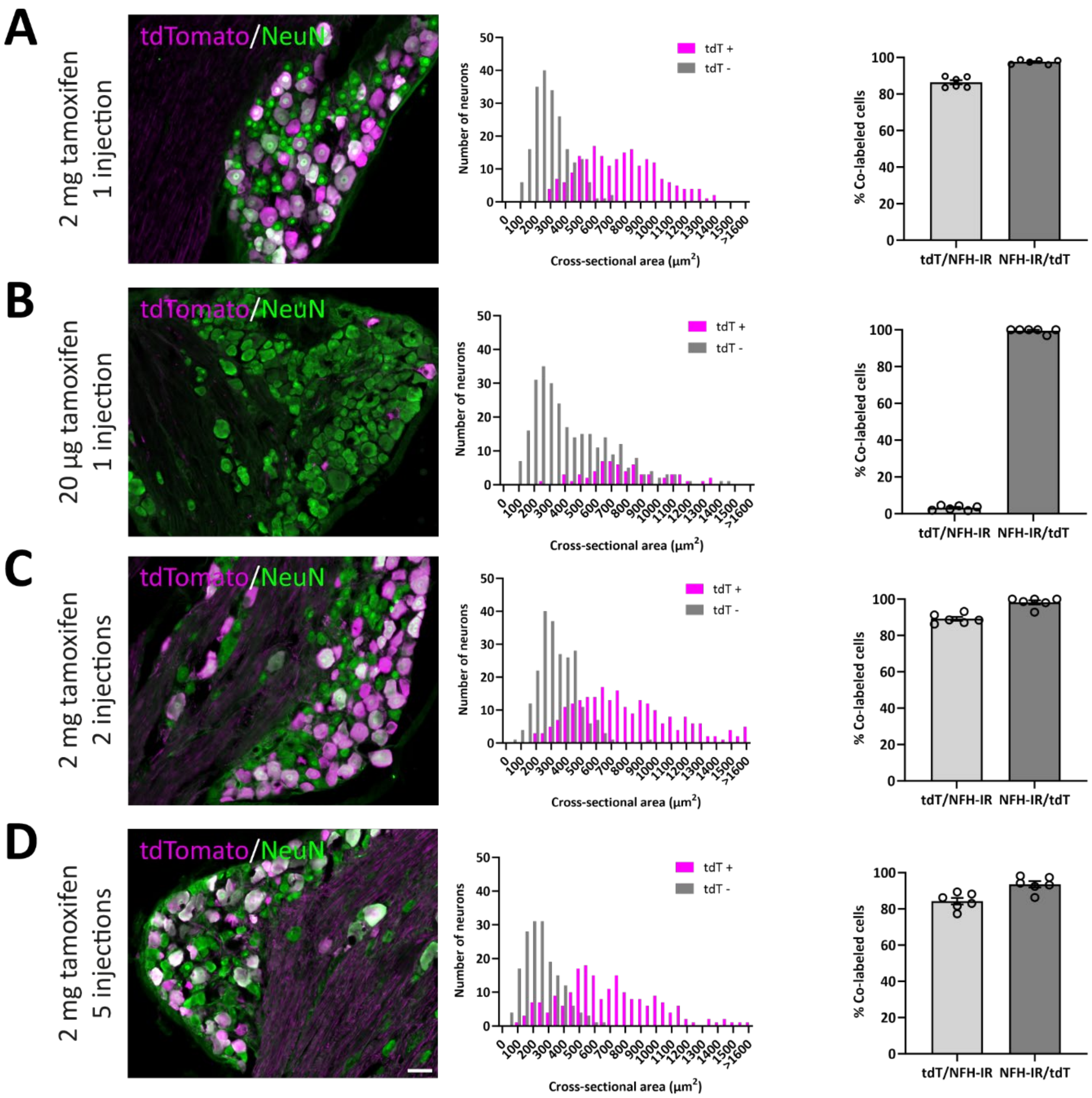

Figure S1. Soma size and recombination efficiency in lumbar DRGs after different tamoxifen administration regimes. **A**, a single low-dose (20 µg) tamoxifen injection. **B-D**, one (B), two (C) or five (D) injections of 2 mg tamoxifen. Left panels, representative images of DRGs showing tdTomato expression among NeuN immunoreactive neurons. Middle panels, size distributions of tdTomato^{+} and tdTomato^{-} DRG neurons. Right panels, recombination efficiency (mean ± S.E.M). with respect to NFH-IR. n=3 mice. Scale bar in D is 50 µm, valid for all panels.
